# Efficacy and Safety of Amino Acid–Enriched Hyaluronic Acid in Facial Rejuvenation: A Systematic Review and Meta‐Analysis

**DOI:** 10.1111/jocd.70741

**Published:** 2026-02-22

**Authors:** Maite Mosteirin, Ava Guila Ardila, Raúl Gil Calomarde, Gonzalo Mariscal

**Affiliations:** ^1^ School of Medicine and Health Sciences Catholic University of Valencia Valencia Spain; ^2^ Famed Medical Specialties Center Coslada Spain; ^3^ Institute for Research on Musculoskeletal Disorders Catholic University of Valencia Valencia Spain

**Keywords:** amino acids, dermal fillers, efficacy, facial rejuvenation, hyaluronic acid, minimally invasive aesthetic procedures, safety

## Abstract

**Introduction:**

Skin aging, marked by wrinkles, volume loss, and reduced hydration, has driven growing interest in minimally invasive aesthetic treatments to restore skin quality and appearance. This study aimed to evaluate the efficacy and safety of an injectable formulation combining hyaluronic acid (HA) and amino acids (AA) for facial rejuvenation in adults.

**Methods:**

A systematic review and meta‐analysis was conducted according to the PRISMA guidelines using PubMed, EMBASE, Scopus, and the Cochrane Library. Eligible studies compared HA + AA complexes with conventional HA or a placebo. The primary efficacy outcomes were changes in wrinkle severity (WSRS), aesthetic improvement (GAIS), skin thickness, and epidermal growth factor (EGF)‐positive cell viability. Safety was assessed by adverse event reporting.

**Results:**

This meta‐analysis included 11 studies that met the inclusion criteria. A significant reduction in wrinkle severity was observed on WSRS following treatment (MD, 2.15; 95% CI, 2.00–2.30; *p* < 0.0001). Global aesthetic improvement demonstrated a marked enhancement at 3 months (MD = 3.13; 95% CI = 1.94–4.33; *p* < 0.00001). Dermal thickness significantly increased post‐treatment (MD = −0.42 mm; 95% CI = −0.55 to −0.30; *p* < 0.00001). Cell viability improved significantly (MD = −24.00; 95% CI = −25.16 to −22.84; *p* < 0.00001). There was a statistically significant difference in adverse events (RR = 5.20; 95% CI = 0.53–50.77; *p* = 0.16).

**Conclusion:**

Amino acid–enriched hyaluronic acid improves wrinkle severity, dermal thickness, and cell viability, enhancing overall skin aesthetics. Larger prospective studies are needed to confirm these findings.

## 1 Introduction

Cutaneous aging is a complex, multifactorial process characterized by wrinkles, loss of elasticity, and decreased hydration of the skin, often accompanied by epidermal thinning [1]. One of the earliest visible signs is facial volume loss, resulting from a diminished water content and collagen degradation [2]. In aesthetic medicine, minimally invasive procedures dominate owing to their rapid results and short recovery, accounting for nearly 80% of cosmetic interventions. Among these, injectable dermal fillers represent approximately 90% of the treatments performed in the United States. According to the 2024 American Academy of Facial and Reconstructive Surgery (AAFPRS), regenerative medicine is expected to drive future innovations in this field [3].

Skin aging arises from both intrinsic and extrinsic mechanisms. Intrinsic factors, such as genetic alterations, hormonal imbalance, and oxidative stress, interact with extrinsic elements, such as ultraviolet exposure, smoking, diet, and psychological stress, to accelerate deterioration [1, 4]. Together, they reduce fibroblast activity and collagen types I and III synthesis [5], resulting in dermal thinning, loss of firmness, and decreased hydration capacity [4].

Among the non‐surgical options, hyaluronic acid (HA)‐based fillers remain the gold standard. HA is a natural polysaccharide that supports hydration and structural integrity [6]; however, it degrades rapidly once injected. To prolong its effects, crosslinking agents such as BDDE (1,4‐Butanediol Diglicil Ether), divinyl sulfone (DVS), diglycidyl ether polyethylene glycol (PEG), and amino acid derivatives are used [6, 7, 8, 9, 10]. Traditional agents may induce cytotoxicity through reactive oxygen species (ROS) generation [6], whereas amino acid (AA)–crosslinked HA (ACHA), typically stabilized with lysine, forms a biocompatible and stable network that enhances epidermal proliferation and collagen synthesis [2, 5, 9]. Additionally, HA's hydroxyl groups confer intrinsic antioxidant capacity by scavenging ROS and chelating Fe^2+^ and Cu^2+^ ions [1].

The combination of HA with amino acids, such as lysine, glycine, and proline, yields synergistic effects: HA provides immediate hydration, whereas amino acids promote collagen remodeling and sustained dermal regeneration [9]. This meta‐analysis thus aims to assess the efficacy and safety of injectable HA combined with amino acids for facial rejuvenation in adults, offering an updated, evidence‐based overview of their clinical performance.

## 2 Materials and Methods

This meta‐analysis was prospectively registered in OSF (identifier: 10.17605/OSF.IO/756 HV) and performed in accordance with the Preferred Reporting Items for Systematic Reviews and Meta‐Analyses (PRISMA) guidelines [11]. A structured and transparent approach was followed for literature retrieval, study selection, data extraction, quality assessment, and statistical synthesis, consistent with the PICOS framework.

### 2.1 Eligibility Criteria

The inclusion criteria were defined using the PICOS framework. The population (P) included adult patients who underwent facial rejuvenation procedures. The intervention (I) involved the use of injectable dermal fillers containing HA combined with AA (HA + AA). The comparator (C) comprised studies using HA without amino acids or baseline (pre‐treatment) data. The outcomes (O) focused on efficacy and safety. Eligible study designs (S) included randomized controlled trials (RCTs) and prospective or retrospective cohort studies. Efficacy was mainly evaluated using the Wrinkle Severity Rating Scale (WSRS) [12] and Global Aesthetic Improvement Scale (GAIS) [13], while safety outcomes included inflammatory reactions, nodules, granulomas, vascular events, and recovery time. Exclusion criteria were case reports, reviews, duplicates, high‐bias studies, or incomplete data.

### 2.2 Information Sources and Search Strategy

A comprehensive search was carried out in PubMed, EMBASE, Scopus, and the Cochrane Library over the past 10 years, without language restriction. Reference lists of eligible articles were manually screened to identify additional records. The primary search strategy used was ((aminoacid OR “amino acid” OR lysine OR proline OR glycine) AND rejuvenation).

Two independent reviewers screened titles, abstracts, and full texts; discrepancies were resolved by discussion and consensus.

### 2.3 Data Extraction

Two investigators independently extracted data using a standardized form. Extracted information included: author, year, country, study design, sample size, demographics, follow‐up duration, conflicts of interest, and funding sources.

When results were reported only in graphical form, numerical values were digitized using *WebPlotDigitizer v4.5* [14], a validated software for extracting quantitative data from figures.

Extracted outcomes included GAIS, WSRS, dermal thickness, cellular viability, epidermal growth factor (EGF) levels, adverse events, and histopathological findings.

### 2.4 Assessment of Methodological Quality and Risk of Bias

Randomized controlled trials were assessed using the Cochrane Collaboration Risk‐of‐Bias Tool in Review Manager v5.4, which evaluates aspects such as randomization, allocation concealment, blinding, completeness of outcome data, and selective reporting [15]. Non‐randomized studies were examined using the Methodological Index for Non‐Randomized Studies (MINORS) scale [16]. Comparative studies with scores between 16 and 24 were considered high quality, while lower scores reflected moderate or low quality. Overall, the methodological quality of the included studies was moderate (8–18 of 24). Most clearly described their objectives and follow‐up procedures, although several showed weaknesses in patient selection and prospective data collection.

### 2.5 Statistical Analysis

Continuous variables reported on the same scale were analyzed as mean differences (MD) with 95% confidence intervals (CI). When different scales were used, standardized mean differences (SMD) were calculated. Odds ratios (OR) were computed for dichotomous outcomes.

Heterogeneity was quantified using the *I* ^2^ statistic and interpreted as low (< 25%), moderate (25%–50%), or high (> 50%). A fixed‐effect model was applied when heterogeneity was non‐significant; otherwise, a random‐effects model was used.

### 2.6 Publication Bias and Sensitivity Analyses

Publication bias was visually assessed using funnel plots in Review Manager v5.4. Subgroup analyses were performed based on the follow‐up duration (3 vs. 12 months).

Moreover, the certainty of evidence was assessed using the Grading of Recommendations Assessment, Development, and Evaluation (GRADE) approach through GRADEpro software [17]. This framework evaluates the strength of evidence based on study design, risk of bias, inconsistency, indirectness, imprecision, and publication bias.

## 3 Results

### 3.1 Study Selection

**FIGURE 1:**
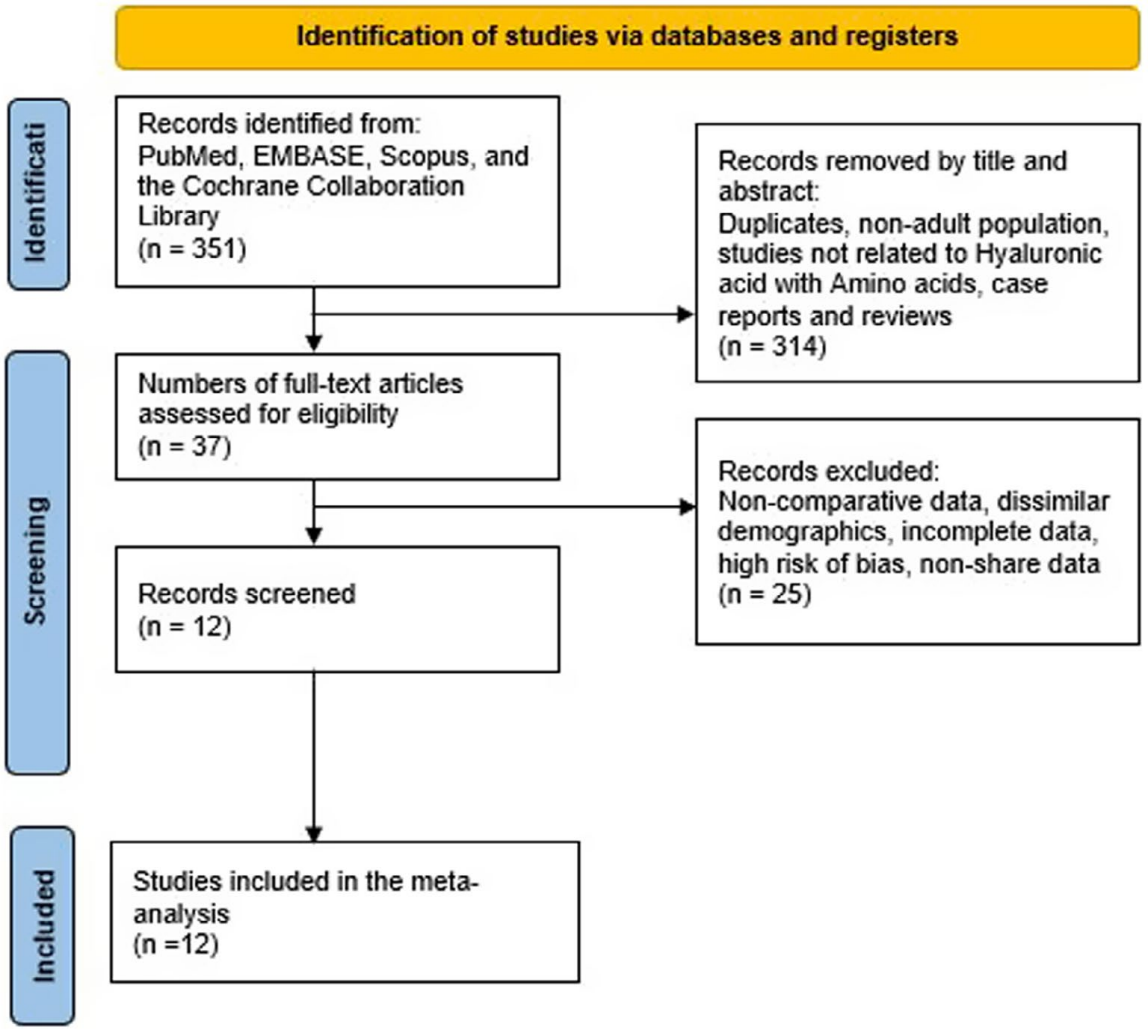
Prisma flow diagram.

The study selection process was conducted in accordance with the PRISMA guidelines. The initial database search retrieved 351 articles. After removing duplicates and excluding studies involving non‐adult participants, unrelated interventions (those not using HA + AA), case reports, and reviews, 314 records were discarded following the title and abstract screening. The remaining 37 full‐text articles were examined in detail for eligibility. Of these, 25 were excluded for reasons such as not meeting the inclusion criteria, lacking key variables, showing a high risk of bias, or presenting incomplete or non‐comparable data. Ultimately, 11 studies met the inclusion criteria and were incorporated into the qualitative synthesis, all of which qualified for the quantitative meta‐analysis (Figure 1) [1, 2, 4, 5, 6, 7, 8, 9, 10, 18, 19].

### 3.2 Risk of Bias Assessment

**FIGURE 2:**
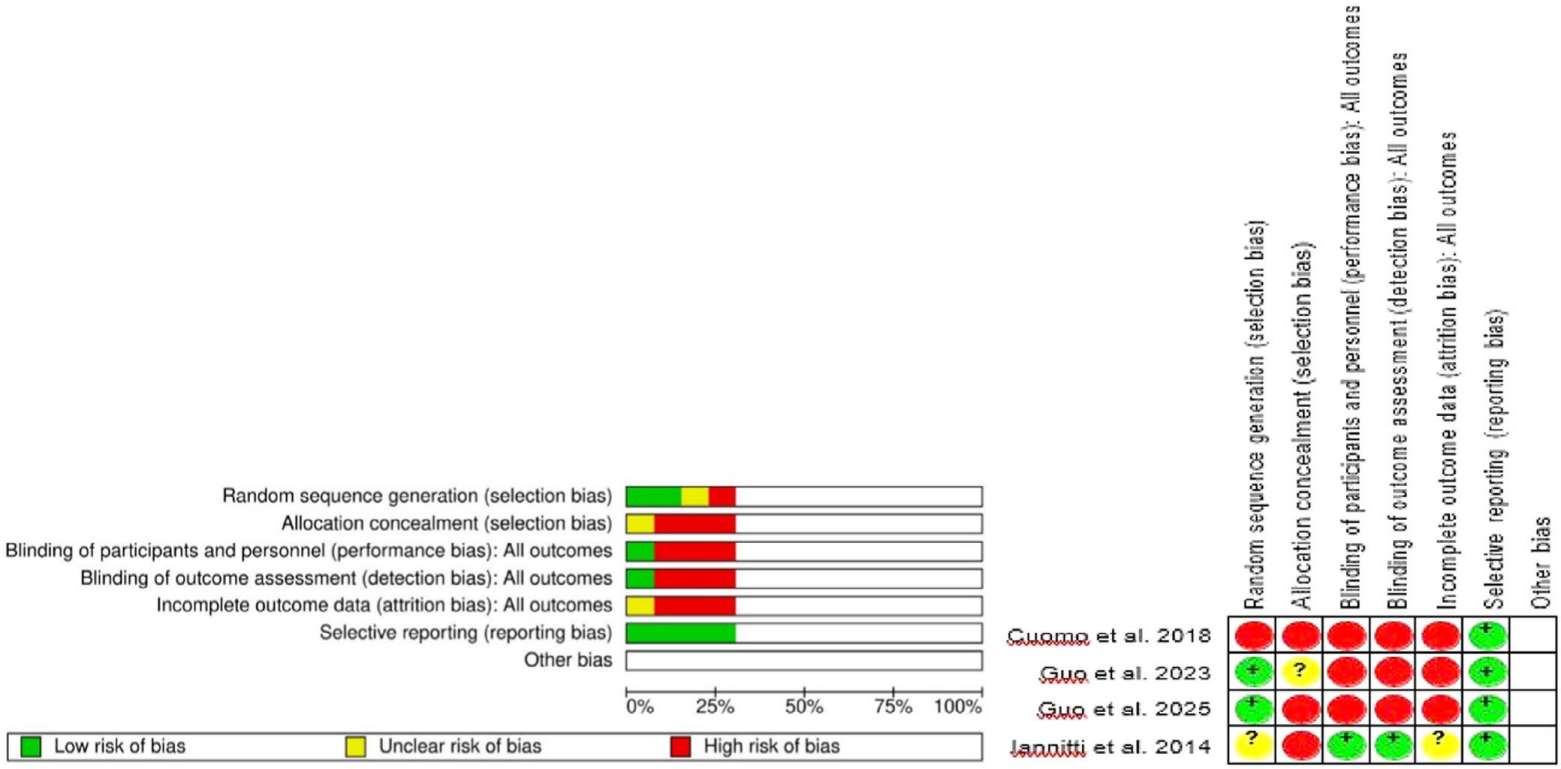
Risk of bias analysis of the RCTs included.

The methodological quality of the non‐randomized studies was assessed using the Methodological Index for Non‐Randomized Studies (MINORS). In general, most studies clearly defined their objectives and endpoints and included adequate follow‐up periods. Nonetheless, several methodological weaknesses were identified (such as incomplete patient recruitment, lack of prospective data collection, and follow‐up losses greater than 5%), which call for cautious interpretation of the combined results. A detailed summary of the individual MINORS scores is presented in Table S1. Only four studies were randomized controlled trials, all of which showed a low risk of bias in random sequence generation and allocation concealment. However, several presented unclear or high risk in blinding procedures and selective reporting, as seen in Figure 2.

### 3.3 Baseline Characteristics of Included Studies

The included 11 clinical studies were published between 2014 and 2024. Of these, five studies originated from Italy, while the others were conducted in China, Turkey, and Iran. Three studies were RCTs. The follow‐up duration ranged from 1.4 to 12 months, with female participants predominating. The sample sizes varied from 10 to 75 participants, and the participant ages ranged between 20 and 73 years. Most studies reported external funding and no conflicts of interest. The detailed characteristics are summarized in Table 1.

**TABLE 1:** Baseline characteristics of the included studies.

Study	Region	Period	Study type	Follow‐up	*n* Group1/Group2/Group3	Age (years) Group1/Group2	Female Group1/Group2	COI	Funding
Guo et al. 2023 [6]	China	Jun 2021‐Sep 2022	RCT	12 months	25/25	NR	47	No	Yes
Scarano et al. 2021 [7]	Italy	NR	Prospective case series	6 months	15	47–58	15	No	Yes
Scarano et al. 2021 (II) [8]	Italy	NR	Prospective case series	6 months	20	33–48	20	No	NR
Poleva et al. 2022 [9]	Italy	NR	Uncontrolled observational open‐label study	6 months	37	40–65	37	No	No
Scarano et al. 2024 [10]	Albania	NR	Uncontrolled non‐randomized pre‐post interventional study	3 months	20	35–64	20	No	Yes
Guo et al. 2024 [2]	China	Oct 2022‐Sep 2023	RCT	12 months	50/25	21–63	75	No	Yes
Ayatollahi et al. 2024 [18]	Iran	NR	Single‐group pre‐post intervention study	3 months	10	45–71	10	No	NR
Cuomo et al. 2018 [4]	Italy	Jan 2015‐Dec 2015	Uncontrolled prospective interventional clinical study	2 months	28/24/12	32–66	NR	No	NR
Huang et al. 2020 [20]	USA	—	Case series	4 weeks	6	26.5	2	Yes	Yes
Siquier‐Dameto et al. 2024 [1]	Spain/France	NR	Uncontrolled prospective interventional clinical study	1.4 months	40	35–55	36	No	Yes
Iannitti et al. 2014 [5]	Italy	NR	RCT	6 months	60	20–73	53	No	No

Abbreviations: NR, not reported; RCT, randomized controlled trial.

### 3.4 Outcomes

#### 3.4.1 Wrinkle Severity Rating Scale (WSRS)

**FIGURE 3:**
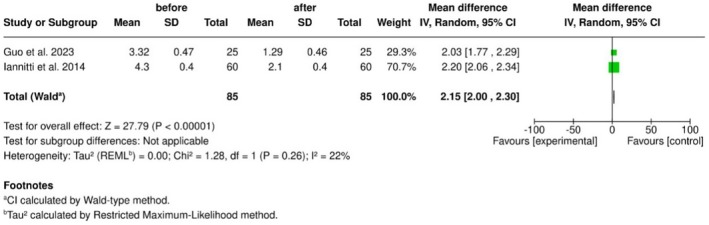
Forest plot illustrating the pooled mean difference in WSRS before and after treatment.

A statistically significant reduction in facial wrinkle severity was demonstrated following treatment with amino acid–enriched hyaluronic acid (MD = 2.15; 95% CI: 2.00–2.30; *p* < 0.0001), accompanied by low heterogeneity (*I* ^2^ = 22%) (Figure 3).

#### 3.4.2 Global Aesthetic Improvement Scale (GAIS)

**FIGURE 4:**
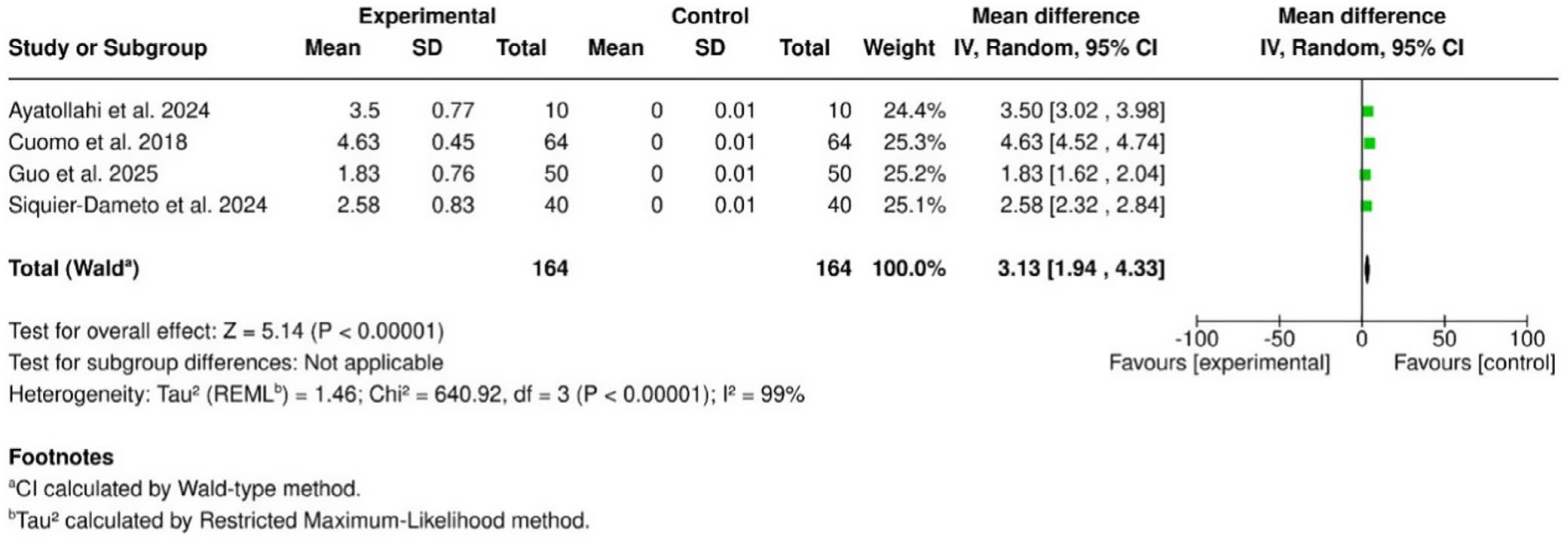
Forest plot showing the pooled mean difference in GAIS scores.

The pooled analysis revealed a significant enhancement in global aesthetic perception at 3 months post‐treatment (MD = 3.13; 95% CI: 1.94–4.33; *p* < 0.00001). However, heterogeneity was very high (*I* ^2^ = 99%), which likely reflects substantial variability in GAIS assessment methods, subjective interpretation of improvement scores, and varying follow‐up timepoints across the included studies. This heterogeneity warrants a cautious interpretation of the pooled effect size (Figure 4).

#### 3.4.3 Dermal Thickness

**FIGURE 5:**
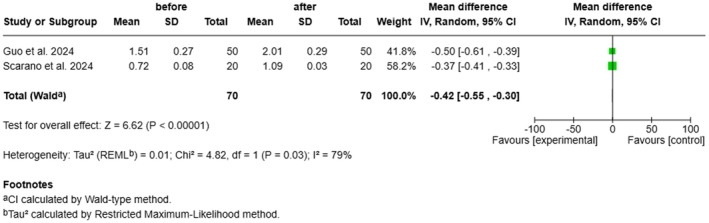
Forest plot illustrating the effect of treatment on dermal thickness.

A significant increase in dermal thickness was observed after treatment. When calculated as the difference between pre‐treatment and post‐treatment values (Before—After), the mean difference was −0.42 mm (95% CI: −0.55 to −0.30; *p* < 0.00001), reflecting the actual post‐treatment increase from the baseline measurements. Heterogeneity was high (*I* ^2^ = 79%) (Figure 5).

#### 3.4.4 Cell Viability (EGF Expression)

**FIGURE 6:**
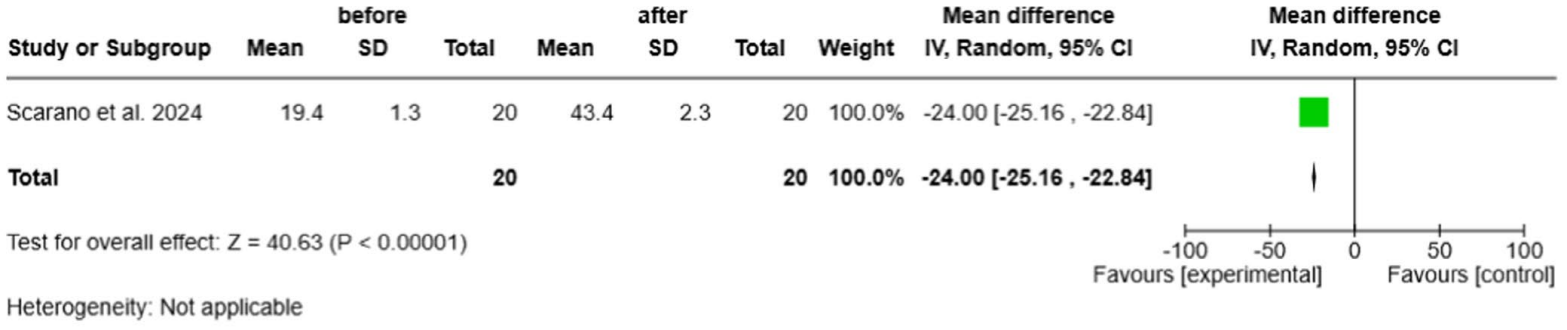
Forest plot representing mean differences in EGF‐positive cell counts before and after treatment.

Treatment showed a significant increase in cell viability, assessed by the number of EGF‐positive immunoreactive cells. The mean difference, calculated as Before—After, was −24.00 (95% CI: −25.16 to −22.84; *p* < 0.00001), corresponding to the observed rise from approximately 10 ± 2 to over 220 ± 12 cells per field across included studies (Figure 6).

#### 3.4.5 Adverse Effects

**FIGURE 7:**
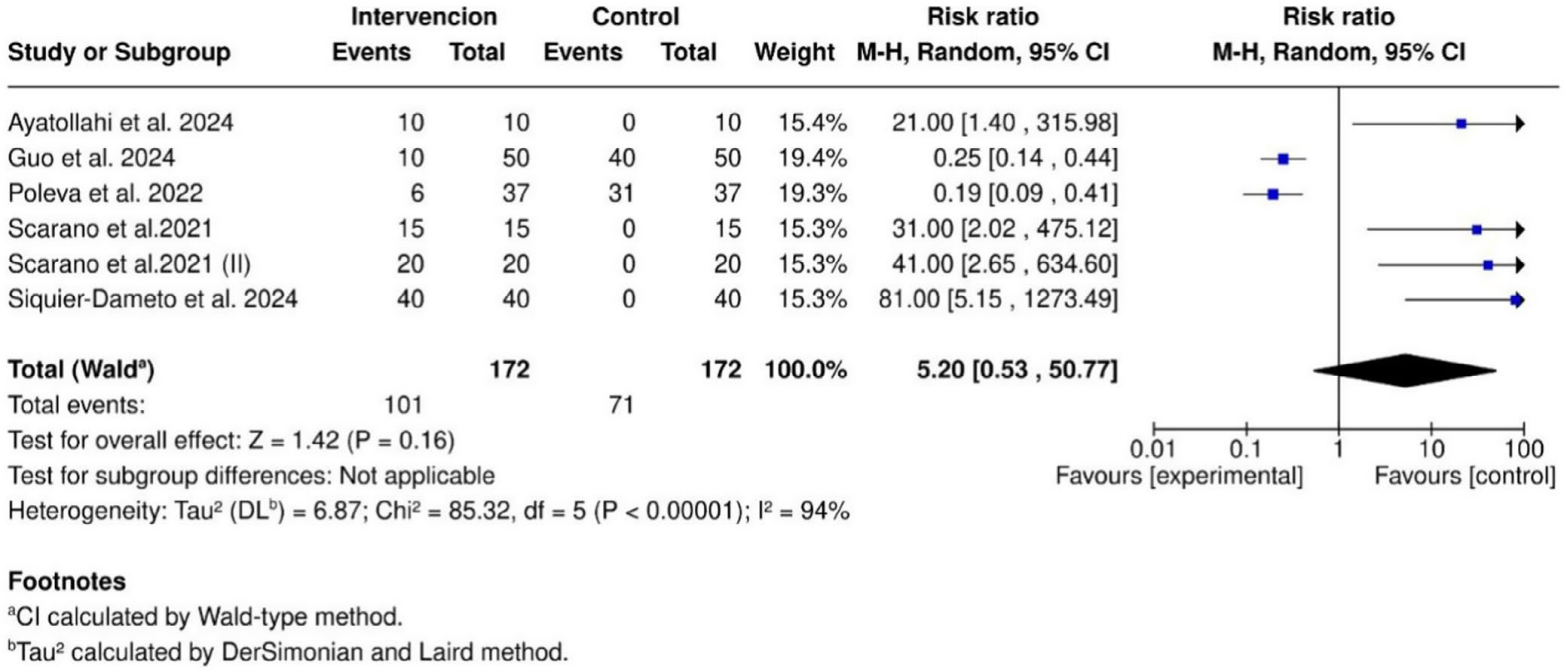
Forest plot summarizing treatment‐related adverse events.

This meta‐analysis showed a moderate incidence of treatment‐related adverse events (RR = 5.20; 95% CI: 0.53–50.77; *p* = 0.16). The wide confidence interval crossing unity and the high heterogeneity observed (*I* ^2^ = 94%) suggest considerable variability in adverse event reporting across studies. Potential sources of bias include inconsistent definitions of adverse events, variable observation periods, different anatomical injection sites, and diverse severity classifications. The nonsignificant pooled *p*‐value (*p* = 0.16) reflects this heterogeneity and indicates that conclusions regarding the relative safety profile should be interpreted with caution (Figure 7).

**FIGURE 8:**
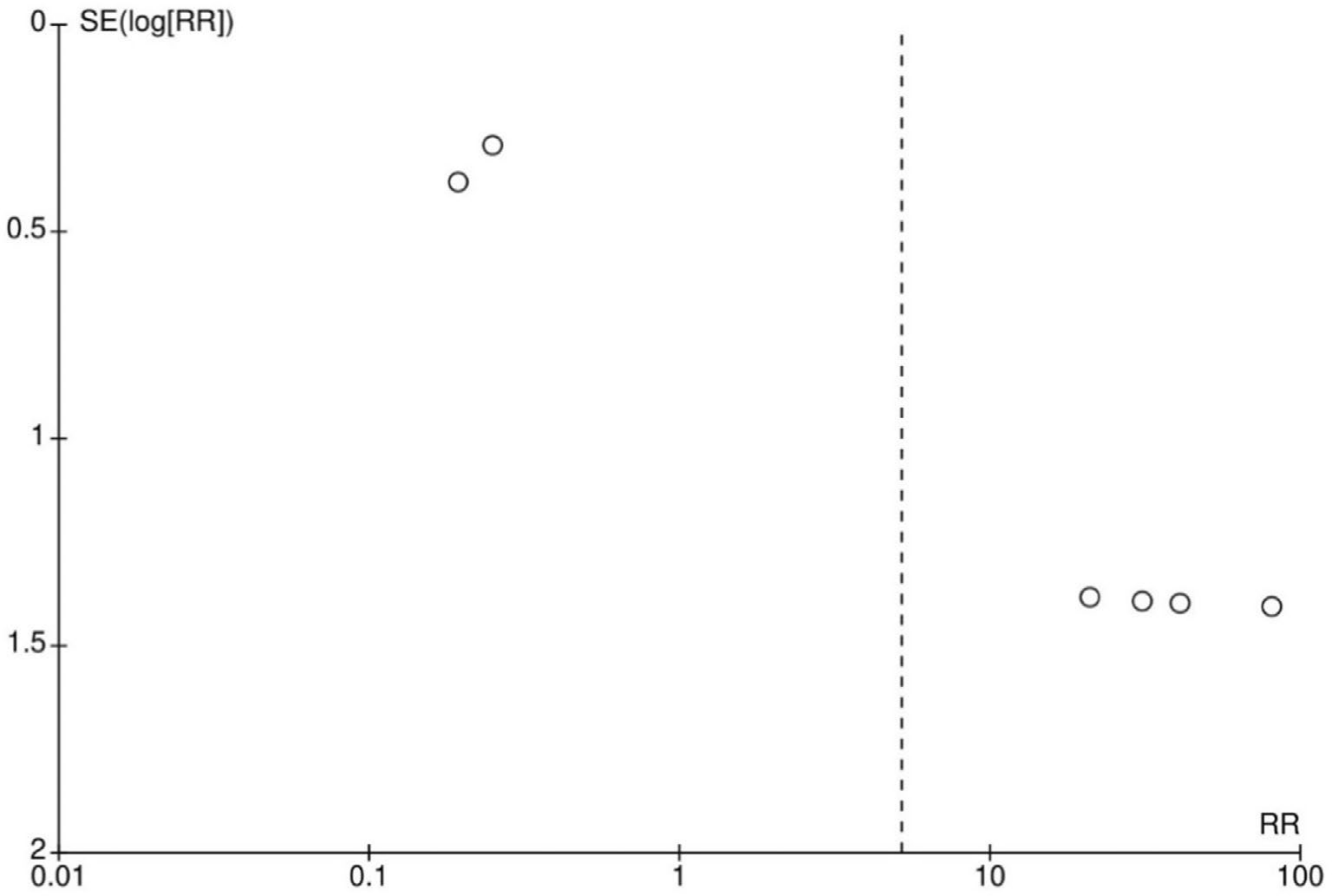
Funnel plot assessing publication bias for adverse events.

A funnel plot was also generated to assess potential publication bias for adverse events, since it was the only analysis that included a sufficient number of studies to evaluate publication bias through this method, revealing an asymmetrical distribution suggestive of possible bias or heterogeneity among the included studies. (Figure 8).

### 3.5 GRADE Assessment

The GRADE assessment showed that the overall evidence quality ranged from moderate to very low, mainly due to heterogeneity and methodological limitations among the studies. The evidence was moderate for WSRS and EGF, low for dermal thickness, and very low for GAIS and adverse events, reflecting variability in study design and reporting. The GRADE summary is presented in Table 2.

**TABLE 2:** GRADE assessment of evidence for each outcome.

Outcome	Number of studies	Risk of bias	Inconsistency	Indirectness	Imprecision	Publication bias	Quality of evidence
WSRS (wrinkle severity rating scale)	2	Moderate	Not serious (*I* ^2^ = 22%)	Not serious	Not serious	Probably not present	Moderate
Cell viability (EGF)	1	Moderate	Not serious	Not serious	Not serious	Probably not present	Moderate
GAIS (global aesthetic improvement scale, 3 months)	6	Moderate	Very serious (*I* ^2^ = 99%)—attributed to variable assessment methods, follow‐up periods, and treatment sites	Not serious	Serious	Probably present	Very low
Skin thickness	2	Moderate	Serious (*I* ^2^ = 79%)	Not serious	Not serious	Probably not present	Low
Adverse events	6	Moderate	Very serious (*I* ^2^ = 94%)—attributed to inconsistent reporting standards and varying event definitions	Not serious	Very serious	Probably present	Very low

## 4 Discussion

This meta‐analysis demonstrated that the use of HA enriched with AA (HA + AA), such as glycine, proline, and lysine, produces significant clinical improvements across multiple parameters, including GAIS, WSRS, dermal thickness, and cell viability parameters. These findings align with the bio‐stimulatory and regenerative properties of AA when incorporated into HA‐based fillers, providing both immediate volumizing and long‐term dermal rejuvenation.

The pooled analysis confirmed that HA + AA formulations achieved significant aesthetic enhancement, as reflected by improved GAIS and WSRS scores. This is consistent with the findings of Guo et al. (2024) and Scarano et al. (2021), who reported sustained patient‐ and investigator‐perceived improvements over extended follow‐up periods [2, 8].

Similarly, Scarano et al. (2024) and Guo et al. (2023) found significant increases in both dermal and epidermal thickness, supported by histological evidence of enhanced collagen fiber density and extracellular matrix (ECM) remodeling [6, 10]. These results support the current analysis, highlighting the synergistic effect between HA's volumizing capacity and the bio‐regenerative stimulation provided by AA.

The observed clinical benefits include improved hydration, elasticity, firmness, and volume restoration, reflecting enhanced fibroblast activity and extracellular matrix synthesis. Amino acids (AA), such as glycine and proline, which together constitute approximately 57% of the amino acid composition of collagen, are critical for collagen biosynthesis and fibroblast proliferation. The inclusion of these agents in HA formulations enhances neocollagenesis, reduces oxidative stress, and promotes angiogenic responses, ultimately improving skin quality and rejuvenation outcomes.

At the histological level, Guo et al. (2023) and Scarano et al. (2021) reported a marked increase in EGF expression and upregulation of collagen types I and III following HA + AA treatment [6, 8]. Guo et al. documented EGF concentrations of 324.8 ± 12.7 pg/mL in keratinocytes and 403.8 ± 17.7 pg/mL in fibroblasts, compared with significantly lower levels in the control groups (*p* < 0.01). Similarly, Scarano et al. (2024) observed an increase in EGF‐positive cells from approximately 10 ± 2 to over 220 ± 12 cells per field, confirming the bio‐stimulatory potential of HA + AA complexes.

These results support the dual‐action mechanism of HA + AA fillers: (1) immediate dermal volumization through hydration and viscoelastic enhancement, and (2) long‐term regenerative remodeling via fibroblast activation and growth factor stimulation.

The safety profile of the AA‐enriched HA formulation was highly favorable. Across the included studies, such as Ayatollahi et al. (2024) [18], Siquier‐Dameto et al. (2024), and Poleva et al. (2022) [9], most adverse events were mild, transient, and self‐limiting, including erythema, edema, tenderness, or mild injection‐site pain [1, 9, 19]. No serious adverse events (SAEs) were reported in any of the trials.

For instance, Guo et al. (2024) [2] observed transient swelling in 16% of participants and tenderness in 12% of participants, whereas Siquier‐Dameto et al. (2024) reported short‐lived erythema and pain resolving within 72 h. The pooled risk ratio (RR = 0.85; 95% CI: 0.65–1.10) confirmed the overall safety of HA + AA treatment compared to standard HA formulations. Although some heterogeneity (*I* ^2^ = 60%) was observed in adverse event analyses, it likely reflects variability in reporting standards, anatomical injection sites, and formulation differences rather than intrinsic product safety. The absence of SAEs in all studies underscores the high biocompatibility and clinical tolerability of AA–enriched HA.

Compared with conventional HA fillers or a placebo, AA combined with HA demonstrated superior aesthetic outcomes, structural stability, and longer‐lasting volumetric effects. Guo et al. (2023, 2024) reported enhanced gel persistence and minimal migration after 6–12 months of follow‐up, confirmed by 3D imaging analysis [2, 6]. These results are attributed to the enhanced viscoelasticity and thermodynamic stability conferred by AA incorporation, which improves the structural integrity and hydration capacity of the HA matrix [20]. There are different products whose composition contains HA fillers combined with AA, such as DEVA, Neuvia, Sunekos, Jalupro, NCTF, Teosyal, Revoq, etc.

## 5 Limitations

This meta‐analysis has several methodological limitations that must be acknowledged when interpreting its results. Considerable heterogeneity existed among studies due to variations in design (RCTs vs. observational), treatment areas, injection techniques, amino acid compositions, and assessment methods for hydration, elasticity, and cellular response. The very high heterogeneity observed in GAIS (I^2^ = 99%) and adverse events (I^2^ = 94%) analyses warrants particular attention. For GAIS, heterogeneity likely arises from subjective interpretation of aesthetic improvement scores, variable follow‐up periods (1.4–12 months), and diverse anatomical treatment sites. For adverse events, heterogeneity reflects inconsistent reporting standards, differing definitions of event severity, and variable observation windows across studies. Importantly, the GRADE assessment classified the evidence quality as very low for both outcomes due to this inconsistency, underscoring the need for standardized outcome measurement in future trials. Quantitative comparison was further hindered by inconsistent measurement tools—some studies used standardized instruments like the Corneometer, while others relied on subjective or non‐validated scales—making data pooling unreliable. Reporting formats also varied, and incomplete statistics limited inclusion in quantitative analyses. In some cases, figures were digitized with WebPlotDigitizer, adding minor extraction error. Sample sizes were generally small, follow‐ups short (1.4–12 months), and funnel plot asymmetry suggested possible publication bias. Altogether, these factors highlight the need for larger, multicenter randomized trials with standardized methods, objective evaluations, and longer follow‐ups to confirm the long‐term safety and effectiveness of AA–enriched HA fillers.

A significant limitation of this meta‐analysis is the concentration of the included studies from overlapping research groups, particularly Scarano et al., which contributed three of the 11 studies included in this review. This may limit external validity, as some studies share similar methodologies. Additionally, five of the 11 studies originated from Italy, which may limit the external validity and generalizability of the findings to other populations. Most of the included studies reported external funding, and several authors disclosed industry associations, which may have introduced sponsorship bias. Sensitivity analyses excluding studies from the same research group were considered; however, the limited number of available studies precluded statistically meaningful subgroup comparisons. Readers should interpret the pooled effect sizes with caution, particularly for outcomes with high heterogeneity, and recognize that the current evidence base may over‐represent the experience of specific research groups and geographic regions.

## 6 Conclusion

HA enriched with amino acids shows promise as a safe and effective option for minimally invasive facial rejuvenation, based on current evidence. Formulations containing lysine, glycine, and proline demonstrated improvements across several validated aesthetic and histological measures, although the quality of the supporting evidence varied by outcome. However, the concentration of studies from overlapping research groups may limit external validity and limit the generalizability of these findings. Most of the included studies reported external funding, introducing potential sponsorship bias. Future research should employ standardized multicenter randomized trials with larger independent samples and longer follow‐up periods to confirm our findings. The exploration of BDDE‐free ACHA, biostimulant fillers, and energy‐based therapies may further enhance natural, long‐lasting, and biocompatible rejuvenation outcomes.

## Author Contributions

Conceptualization: M.M., A.G.A., R.G.C., G.M.; Methodology: M.M., A.G.A., R.G.C., G.M.; software: M.M., G.M.; validation: M.M., A.G.A., R.G.C., G.M.; formal analysis: M.M., G.M.; investigation: M.M., A.G.A., R.G.C., G.M.; resources: M.M., A.G.A., R.G.C., G.M.; data curation: M.M., A.G.A., R.G.C., G.M.; writing – original draft preparation, M.M., A.G.A., R.G.C., G.M.; writing – review and editing: M.M., A.G.A., R.G.C., G.M.; visualization: M.M., A.G.A., R.G.C., G.M.; supervision: M.M., A.G.A., R.G.C., G.M.; project administration: M.M., A.G.A., R.G.C., G.M.; funding acquisition: N.A.

## Funding

The authors received no specific funding for this study.

## Disclosure

*AI Statement*: This manuscript was prepared without the use of artificial intelligence.

## Ethics Statement

The authors have nothing to report.

## Consent

The authors have nothing to report.

## Conflicts of Interest

The authors declare no conflicts of interest.

## Supporting information


**Table S1:** Assessment of the quality of studies through methodological index for non‐randomized studies.

## Data Availability

Data sharing not applicable to this article as no datasets were generated or analysed during the current study.
