# Supplementary material for: Efficacy and Safety of Amino Acid–Enriched Hyaluronic Acid in Facial Rejuvenation: A Systematic Review and Meta‐Analysis
Source: J Cosmet Dermatol. 2026 Feb 22;25(3):e70741. doi: 10.1111/jocd.70741 (PMC12926521; doi:10.1111/jocd.70741)
Supplement: Supplementary file 1 — Table S1: Assessment of the quality of studies through methodological index for non‐randomized studies. [file JOCD-25-e70741-s001.docx]

| **Study** | **Clearly stated aim** | **Consecutive patients** | **Prospective collection data** | **Endpoints** | **Assessment endpoint** | **Follow-up period** | **Loss less than 5%** | **Study size** | **Adequate control group** | **Contemporary group** | **Baseline control** | **Statistical analyses** | **MINORS** |
| --- | --- | --- | --- | --- | --- | --- | --- | --- | --- | --- | --- | --- | --- |
| **Scarano et al. 2021 [7]** | 2 | 0 | 0 | 2 | 2 | 2 | 0 | 1 | - | - | - | - | 10 |
| **Scarano et al. (II)2021 [8]** | 2 | 0 | 0 | 2 | 2 | 2 | 2 | 2 | - | - | - | - | 12 |
| **Scarano et al. 2024 [10]** | 2 | 0 | 0 | 2 | 2 | 1 | 0 | 2 | - | - | - | - | 9 |
| **Ayatollahi et al. 2024 [19]** | 2 | 0 | 0 | 2 | 2 | 1 | 0 | 1 | - | - | - | - | 8 |
| **Poleva et al. 2022 [9]** | 2 | 0 | 0 | 2 | 2 | 2 | 2 | 2 | - | - | . | - | 12 |
| **Siquier-Dameto et al. 2024 [1]** | 2 | 0 | 0 | 2 | 2 | 2 | 0 | 2 | - | - | - | - | 10 |
| **Huang et al. 2020 [20]** | 2 | 0 | 0 | 2 | 2 | 2 | 0 | 1 |  |  |  |  | 9 |

Table S1: Assessment of the quality of studies through Methodological Index for Non-Randomized Studies.
